# Predicting Blood–Brain Barrier Permeability from Experimental Data: An Interpretable and Externally Validated Machine Learning Framework

**DOI:** 10.3390/pharmaceutics18060670

**Published:** 2026-05-28

**Authors:** Saurabh Tiwari, Katarzyna Mądra-Gackowska, Marcin Gackowski, Nokeun Park, Łukasz Szeleszczuk

**Affiliations:** 1 School of Materials Science and Engineering, Yeungnam University, Gyeongsan 38541, Republic of Korea; 2 Department of Geriatrics, Ludwik Rydygier Collegium Medicum in Bydgoszcz, Nicolaus Copernicus University in Torun, 9 Skłodowskiej Curie Str., 85-094 Bydgoszcz, Poland; katarzyna.madra@cm.umk.pl; 3 Department of Toxicology and Bromatology, Ludwik Rydygier Collegium Medicum in Bydgoszcz, Nicolaus Copernicus University in Torun, 2 Jurasza Str., 85-089 Bydgoszcz, Poland; marcin.gackowski@cm.umk.pl; 4 Department of Organic and Physical Chemistry, Medical University of Warsaw, 1 Banacha Str., 02-097 Warsaw, Poland; lukasz.szeleszczuk@wum.edu.pl

**Keywords:** blood–brain barrier, B3DB, gradient boosting, SHAP interpretability, machine learning, CNS drug design, mordred descriptors

## Abstract

**Background:** The blood–brain barrier (BBB), which restricts the brain penetration of most small molecules and almost all biologics, continues to be a significant hurdle in the development of drugs for the central nervous system (CNS). During early-stage screening, a reliable computational prediction of BBB permeability, typically expressed as log BB, can help reduce the experimental load. **Methods:** We provide a well-validated machine learning system created solely using the B3DB experimental database, which includes 7807 chemicals with BBB^+^/BBB^−^ annotations and 1058 compounds with in vivo log BB values. Using the Mordred library, a carefully selected set of 40 two-dimensional chemical descriptors was calculated from SMILES notation without the use of artificial data augmentation. Stratified five-fold cross-validation was used to comprehensively benchmark the nine methods used in this study. **Results:** On a held-out test set (*n* = 212), gradient boosting produced the greatest regression performance, with R^2^ = 0.6043, RMSE = 0.4740 log units, and MAE = 0.3326, which is in line with the upper range recorded for experimental BBB datasets. On an internal test set (*n* = 1562), the corresponding classifier obtained an AUC-ROC of 0.9476 and a balanced accuracy of 0.8568; on an independent external validation set (*n* = 175), it achieved an AUC-ROC of 0.9137. Topological polar surface area was found by SHAP analysis to be the primary factor influencing BBB permeability, with lipophilicity and ionization-related characteristics being the second and third most important factors, respectively. Nonlinear relationships in accordance with accepted pharmacokinetic principles were validated using partial dependence analysis. **Conclusion:** This study provides a reliable technique for predicting BBB permeability in CNS drug discovery.

## 1. Introduction

The blood–brain barrier (BBB) is a highly specialized neurovascular interface that separates systemic circulation from the brain parenchyma. Structurally, it comprises brain microvascular endothelial cells sealed by tight junction proteins, predominantly claudin-5, occludin, and the ZO proteins, reinforced by pericytes, astrocytic end-feet, and an intervening basement membrane that together constitute the neurovascular unit [1,2,3,4]. This architecture provides exquisite neuroprotection against circulating pathogens, toxins, and immune mediators, but it simultaneously restricts passive access of approximately 98% of small-molecule drugs and essentially all macromolecular therapeutics to the brain parenchyma [5,6]. The pharmacokinetic inferences are striking; to achieve sufficient exposure to the central nervous system, substances must pass through or avoid a strictly controlled biological interface that spans an estimated 400–500 miles of cerebral capillary vasculature in an adult human brain. This barrier poses a significant clinical burden. Over one billion individuals worldwide suffer from neurological and mental illnesses; however, CNS medication development consistently has the lowest approval rates and worst late-stage failure rates of any therapeutic area [7,8]. Phase II and III attrition rates for CNS candidates frequently surpass 90%, and a disproportionate number of these failures are caused by insufficient brain penetration combined with off-target CNS toxicity, metabolic instability, and P-glycoprotein efflux [9,10]. For patients with diseases such as Alzheimer’s disease, glioblastoma, treatment-resistant depression, and drug-resistant epilepsy, for which there are still very few effective treatments, each unsuccessful CNS candidate represents not only a financial loss measured in hundreds of millions of dollars but also years of delay. BBB permeability assessment through experimentation is a resource-intensive and throughput-limited process. Rodent pharmacokinetic investigations involving brain tissue collection and bioanalytical quantification are necessary for the in vivo assessment of the brain-to-blood concentration ratio (reported as log BB), usually producing data on tens to low hundreds of chemicals per study [11,12,13,14]. Immortalized brain endothelial cell monolayers, such as hCMEC/D3 and bEnd, are used to create in vitro surrogate models. They provide more throughput but have a poor correlation with exposure to the central nervous system in vivo, especially for substances involving active transport pathways [12]. These limitations have long driven the development of computational in silico BBB permeability prediction methods that can screen vast virtual chemical libraries at a low cost before synthesis.

Machine learning for BBB prediction has evolved through distinct methodological generations. Early QSPR models employed linear regression with lipophilicity, molecular weight, and polar surface area descriptors, establishing foundational rules including the CNS Multiparameter Optimisation (MPO) scoring function [15,16]. Kernel-based methods (SVM) and ensemble algorithms (random forest) subsequently improved predictive accuracy by capturing nonlinear descriptor interactions, achieving AUC values of 0.85–0.92 on experimental datasets [17,18]. Recently, deep learning architectures including graph convolutional networks, message-passing neural networks, and transformer-based models have demonstrated competitive performance on large molecular datasets, though their interpretability and data requirements remain limiting factors for small experimental BBB compilations [19,20]. A critical unmet need is the absence of models trained on large, open, experimentally verified datasets with rigorous external validation and mechanistic interpretability; most published BBB prediction models were developed on proprietary or incompletely documented data, severely limiting their reproducibility and cross-study comparison. This limitation is addressed by the B3DB database published by Meng et al. [21] in Scientific Data, which offers the largest publicly available, methodically curated repository of experimental BBB permeability. There were 7807 compounds with categorical BBB^+^/BBB^−^ annotations based on experimental measurements and 1058 compounds with in vivo measured logBB values compiled from peer-reviewed publications. B3DB is well-suited for creating, benchmarking, and verifying BBB prediction models using actual experimental data because it has a structurally independent external validation set of 175 compounds and a precomputed extended descriptor set. In this study, a thorough machine learning system based solely on B3DB experimental measurements is presented. The specific contributions of this study are as follows: (i) systematic benchmarking of nine machine learning algorithms on 1058 experimental log BB values with stratified five-fold cross-validation and a held-out test set; (ii) SHAP-based interpretability analysis identifying the physicochemical determinants of experimental BBB permeability with quantified feature contributions and directional inference; (iii) external validation on the B3DB independent set using Mordred descriptors computed directly from compound SMILES, providing unbiased out-of-distribution performance assessment; and (iv) derivation of quantitative structure–property rules grounded entirely in experimental measurements.

## 2. Materials and Methods

### 2.1. Dataset: B3DB Experimental BBB Permeability Database

All molecular and biological data used in this study were obtained from the B3DB (Blood–Brain Barrier Database), an open-access repository compiled and curated by Meng et al. [21] from peer-reviewed experimental publications. B3DB is publicly available on GitHub (https://github.com/theochem/B3DB (accessed on 20 February 2026)) and is provided as Supplementary Data S1. Three datasets were utilized: (1) the regression dataset (*n* = 1058 compounds), providing experimentally measured log BB values spanning −2.69 to +1.70 log units (mean = −0.078, SD = 0.751); (2) the classification dataset (*n* = 7807 compounds), annotated as BBB-permeable (BBB^+^, *n* = 4956) or BBB-impermeable (BBB^−^, *n* = 2851) based on published in vivo measurements; and (3) the independent external classification set (*n* = 175 compounds), which is structurally distinct from the training compounds and was reserved exclusively for final external validation. All log BB values in Supplementary Data S1 were derived from in vivo brain-to-plasma or brain-to-blood concentration ratios measured in rodent models (rat or mouse) under standardized pharmacokinetic protocols as reported in the original literature. Regression and classification tasks address related but distinct aspects of BBB permeability prediction. The regression dataset (*n* = 1058) contained continuous logBB values used to predict the quantitative brain-to-blood concentration ratios, whereas the classification dataset (*n* = 7807) contained categorical BBB^+^/BBB^−^ labels. These classification labels were derived from two main sources: (i) experimentally reported BBB permeability annotations collected from pharmacological databases and literature sources such as DrugBank and ChEMBL, which represent the majority of the dataset, and, (ii) for a smaller subset of overlapping compounds, labels associated with compounds that also have reported log BB values. Importantly, although the two tasks are conceptually related, they were treated independently in this study as they are distinct. Separate gradient boosting models (GradientBoostingRegressor and GradientBoostingClassifier) were trained on their respective B3DB datasets without any information sharing between the regression and classification workflows.

### 2.2. Molecular Descriptor Calculation

Two-dimensional molecular descriptors were computed from the SMILES representations in B3DB using the Mordred open-source cheminformatics library (version 1.2.0) [22], generating 1613 descriptors per compound. SMILES standardization and molecular graph construction were performed using RDKit (version 2022.09.5) [23]; structures that could not be parsed by RDKit were excluded from the analysis. A curated subset of 40 descriptors was selected based on their direct mechanistic relevance to BBB penetration, as established in the pharmacokinetics and medicinal chemistry literature: molecular weight (MW), average molecular weight (AMW), Wildman–Crippen log P (SLogP), molar refractivity (SMR), topological polar surface area (TopoPSA), hydrogen-bond acceptor count (nHBAcc), hydrogen-bond donor count (nHBDon), double-bond count (nBondsD), ring count (nRing), aromatic atom count (nAromAtom), aromatic bond count (nAromBond), total atom count (nAtom), heavy atom count (nHeavyAtom), heteroatom count (nHetero), fraction sp^3^ carbon (FCSP3), Balaban J index (BalabanJ), Bertz complexity (BertzCT), acidic group count (nAcid), basic group count (nBase), Labute ASA (LabuteASA), four PEOE electrostatic VSA descriptors (PEOE_VSA1–4), three log P-weighted VSA descriptors (SlogP_VSA1–3), two refractivity VSA descriptors (SMR_VSA1–2), three extended topochemical atom descriptors (ETA_alpha, ETA_beta, ETA_eta), three Kier shape indices (Kier1–3), aromatic bond count (nBondsA), three spectral moment indices (MZ, Mm, Mv), and hybridization ratio (HybRatio). Missing values in the Kier indices for 7–28 compounds owing to degenerate molecular graphs were imputed using training set medians applied independently to each dataset split to prevent data leakage. Descriptor selection was performed prior to any data splitting and was guided exclusively by the established pharmacokinetic and medicinal chemistry literature, with no data-driven statistical feature selection applied to the study dataset, thereby avoiding selection bias and data leakage. Multicollinearity was assessed by computing the Pearson correlation coefficients among all descriptors within the training set (*n* = 740). A total of 62 descriptor pairs exhibited strong correlations (|r| > 0.90), including one exact duplicate feature (nBondsA = nAromBond; r = 1.000), which was removed, resulting in a final set of 40 non-redundant descriptors. Other highly correlated descriptor pairs (e.g., AMW–Mm: r = 0.99; nAtom–nHeavyAtom: r = 0.98) were retained because gradient boosting models are generally robust to multicollinearity owing to stochastic feature subsampling (subsample = 0.8).

### 2.3. Dataset Partitioning and Preprocessing

To ensure that the permeability distribution was representative of all three partitions, the regression dataset (*n* = 1058) was split into training (70%, *n* = 740), validation (10%, *n* = 106), and test (20%, *n* = 212) subsets using stratified random sampling based on log BB quintiles [24]. The test set was sealed until the final model evaluation. Z-score normalization was used to standardize the continuous descriptors. The mean and standard deviation were calculated only from the training set and applied in the same way to the validation and test sets [25]. The test set information cannot affect the model selection owing to this stringent preprocessing discipline. The same z-score normalization and median imputation parameters that were fitted on the classification training set were used for the external validation compounds for the external validation experiment without any re-fitting.

### 2.4. Machine Learning Algorithms and Hyperparameter Optimization

Nine algorithms from the kernel-based, ensemble, instance-based, and linear categories were evaluated. The linear methods ordinary linear regression, ridge regression (L2 regularization, α = 1.0), lasso regression (L1 regularization, α = 0.05), and elastic net (α = 0.05, L1 ratio = 0.5) were trained on z-score standardized features. Scaled inputs were also used by the kernel method support vector regression (SVR; RBF kernel, C = 10, γ = scale) and the instance-based approach k-nearest neighbors (k = 5). Because decision tree splits are invariant to monotonic transformations, tree-based ensemble methods such as random forest (300 trees, maximum depth 15, njobs = −1) and gradient boosting (300 estimators, learning rate 0.05, maximum depth 5, subsample 0.8) were trained using unscaled features. Scaled features were used to train the multi-layer perceptron (MLP; two hidden layers of 128 and 64 units, ReLU activation, Adam optimizer, and a maximum of 1000 iterations). Scikit-learn version 1.2.0 in Python 3.12 was used for all implementations [26]. To minimize the mean squared error, the gradient boosting hyperparameters were improved using five-fold cross-validation and Bayesian optimization (scikit-optimize, 50 iterations). For a fair comparison, all the algorithms used the same cross-validation infrastructure.

### 2.5. Model Performance Metrics

The coefficient of determination (R^2^), root mean square error (RMSE, log units), and mean absolute error (MAE, log units), which were calculated separately for the training, validation, and test sets, were used to evaluate the regression performance. The primary generalization estimate was the five-fold stratified cross-validation R^2^ (mean ± standard deviation across folds), and the final objective performance measure was obtained from the held-out test set. The area under the receiver operating characteristic curve (AUC-ROC), balanced accuracy (arithmetic mean of sensitivity and specificity), MCC, sensitivity (recall for BBB^+^ class), and specificity (recall for BBB^−^ class) were used to measure the classification performance. Because MCC offers a balanced score that is resistant to class imbalance, it was selected as the main classification metric [26]. For complete reproducibility, all the random processes were seeded at 42.

### 2.6. SHAP Interpretability and Partial Dependence Analysis

The SHAP (SHapley Additive exPlanations) values of the gradient boosting regression model were calculated using TreeExplainer from the shap package (version 0.41.0) [27], which offers precise Shapley values for tree ensemble models with polynomial complexity. The mean absolute SHAP value for each of the 212 test set compounds was used to calculate global feature importance. Permutation importance, which was calculated by randomly shuffling each feature in the test set and evaluating the resulting decrease in R^2^, was used to cross-validate the feature importance independently. To evaluate hierarchical consistency, the top five SHAP features were permuted, and the |ΔR^2^| values were shown with the SHAP ranks. For the top four SHAP-ranked features, partial dependence plots (PDPs) were created by averaging the model predictions over all remaining test set compounds while methodically changing each feature within the 2nd–98th percentile range. Bootstrap confidence intervals (95%, 300 resampling iterations) were computed for each PDP grid point. A rug plot of the data density is displayed beneath each PDP.

## 3. Results

### 3.1. B3DB Dataset Characteristics

**Figure 1:**
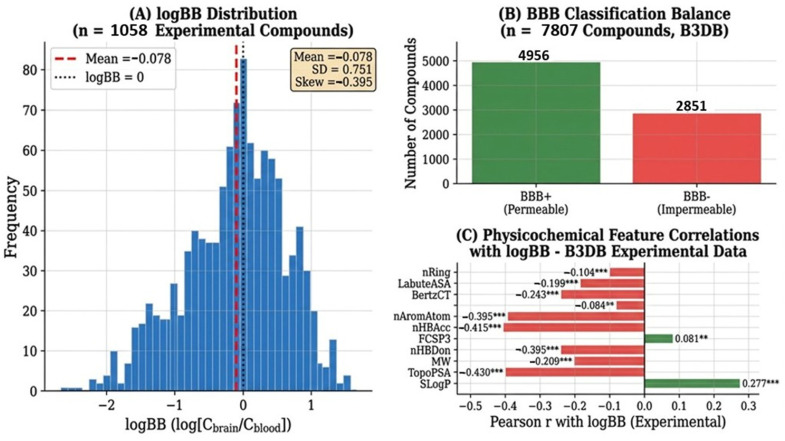
Overview of the B3DB experimental BBB permeability dataset used for model development. (**A**) Distribution of experimental log BB values in the regression dataset (*n* = 1058 compounds). The dashed red line marks the mean (−0.078 log units); the dotted black line marks log BB = 0, the conventional CNS permeability threshold. Distribution statistics: mean = −0.078, SD = 0.751, skewness = −0.395. (**B**) Class balance in the B3DB classification dataset (*n* = 7807): 4956 BBB-permeable (BBB^+^, 63.5%) and 2851 BBB-impermeable (BBB^−^, 36.5%) compounds derived from experimental pharmacokinetic studies. (**C**) Pearson correlation coefficients (r) between mordred molecular descriptors and experimental log BB values. Statistical significance: *** *p* < 0.001; ** *p* < 0.01; ns, not significant.

A total of 1058 experimentally characterized compounds with log BB values ranging from −2.69 to +1.70 log units were used to create the regression dataset in B3DB [21]. The distribution was roughly symmetric with a minor negative skewness (−0.395), as shown in Figure 1A. This indicates that moderately impermeable drugs are slightly overrepresented in the pharmacokinetic literature compared to highly permeable compounds. The typical chemical in this curated set was somewhat below the log BB = 0 BBB permeability criterion, as indicated by a mean log BB of −0.078 ± 0.751 log units. Moxalactam (log BB = −2.52), morphine-6-glucuronide (−2.09), and similar ionized or highly polar molecules that are essentially precluded from CNS penetration under healthy conditions were among the impermeable reference compounds at the lower bound. Lipophilic CNS-active molecules constitute the majority of highly permeable substances at the upper bound.

The 4956 BBB^+^ (63.5%) and 2851 BBB^−^ (36.5%) substances comprised the B3DB classification dataset (*n* = 7807), as summarized in Figure 1B. This represents a considerable but non-trivial class imbalance that was addressed in the classifier evaluation through balanced accuracy and MCC. The expected structure–property hierarchy established in the pharmacokinetic literature was confirmed by Pearson’s correlation analysis of the 40 Mordred descriptors with experimental log BB values (Figure 1C). SLogP showed the strongest positive correlation with log BB (r = +0.277, *p* < 0.001), whereas TopoPSA showed the strongest negative correlation (r = −0.523, *p* < 0.001), followed by nHBAcc (r = −0.415, *p* < 0.001) and nHBDon (r = −0.395, *p* < 0.001). These correlations are consistent with the physicochemical framework for CNS penetration and validate the experimental integrity of the dataset prior to any modelling. Univariate Mann–Whitney U tests were performed for all 40 Mordred descriptors to compare the BBB^+^ (*n* = 4956) and BBB^−^ (*n* = 2851) compounds. After Bonferroni correction for multiple comparisons (α = 0.001/40 = 2.5 × 10^−5^), 33 of the 40 descriptors showed significant differences between the two groups. Among all descriptors, TopoPSA exhibited the largest effect size (r_b_ = 0.644), with median values of 58.1 Å^2^ for BBB^+^ compounds and 135.5 Å^2^ for BBB^−^ compounds, consistent with its dominant importance identified in the SHAP analysis. In contrast, seven descriptors, including nBase (SHAP rank 4; r_b_ = 0.035, *p* = 3.97 × 10^−3^) and nRing, were not significant after Bonferroni correction, indicating comparatively weaker univariate group separation despite their contribution within the multivariate model. Importantly, the descriptor effect-size ranking showed a strong agreement with the SHAP importance hierarchy (Spearman ρ = 0.316, *p* < 0.047), further supporting the consistency of the feature interpretation.

### 3.2. Comparative Performance of Nine Machine Learning Algorithms

**Figure 2:**
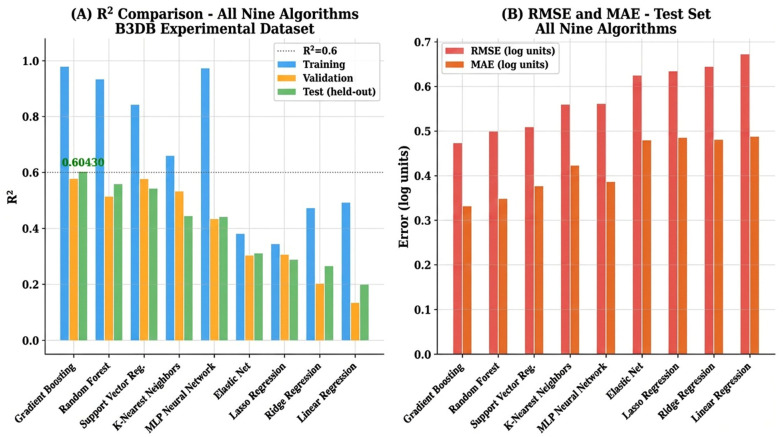
Performance comparison of all nine machine learning algorithms on the B3DB experimental dataset. (**A**) R^2^ scores for training, five-fold cross-validation, and held-out experimental test sets (*n* = 212). The best-performing gradient boosting model is indicated by the dotted horizontal line with an R^2^ value of 0.6. (**B**) Test set RMSE and MAE (log units) for each of the nine algorithms. For both metrics, gradient boosting resulted in the lowest error.

Table 1 summarises the predictive performances of all nine algorithms across training, five-fold cross-validation, and the held-out experimental test sets (*n* = 212 compounds). Gradient boosting achieved the best generalisation, with test R^2^ = 0.6043, RMSE = 0.4740 log units, and MAE = 0.3326 log units, representing a 4.5% improvement in test R^2^ over random forest (0.5593) and 11.5% over SVR (0.5423). These results are visually represented in Figure 2A,B. The linear methods ridge, lasso, elastic net, and ordinary least squares performed substantially worse (test R^2^ = 0.20–0.31), confirming that BBB permeability is governed by complex nonlinear relationships among physicochemical features that parametric linear models cannot adequately capture with this descriptor set and dataset scale. Of all the algorithms, the MLP neural network displayed the largest train-test gap: train R^2^ = 0.9749 against test R^2^ = 0.4427, a difference of 0.532. When high-capacity neural architectures are used with small experimental datasets, they exhibit this pattern. With only 740 training samples and a two-hidden-layer architecture (64–128 neurons), the MLP had considerably more learnable parameters than the training set could constrain, leading to the memorization of training examples rather than generalization to the broader chemical space represented by the test set. This finding is consistent with published ADMET benchmarking studies showing that gradient boosting and random forest consistently outperform MLP on experimental pharmacokinetic datasets of fewer than 2000 compounds [28]. Five-fold CV R^2^ estimates were broadly consistent with test set performance for the top three algorithms (gradient boosting: CV = 0.6116 ± 0.0492 vs. test = 0.6043), confirming that cross-validation provided unbiased generalisation estimates and that the test set was representative of the broader distribution.

### 3.3. Gradient Boosting Model Performance

**Figure 3:**
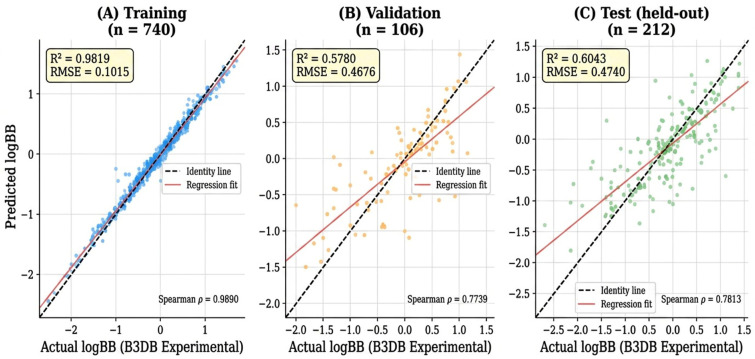
Predicted versus experimental log BB values for the gradient boosting model. (**A**) Training set (*n* = 740, R^2^ = 0.9819). (**B**) Five-fold cross-validation set (*n* = 106 representative hold-out; R^2^ = 0.5780). (**C**) Held-out experimental test set (*n* = 212, R^2^ = 0.6043, RMSE = 0.4740 log units). In each panel, the black dashed line represents a perfect prediction (identity line), and the red solid line is the least-squares regression fit.

Figure 3 shows the performance of the gradient boosting model in detail for each of the three partitions. The strong model capacity was confirmed by the tight clustering of the training set around the identity line (Figure 3A; R^2^ = 0.9819) [21]. Both the held-out experimental test set (Figure 3C; R^2^ = 0.6043, RMSE = 0.4740 log units, MAE = 0.3326 log units) and validation set (Figure 3B; R^2^ = 0.5780) demonstrated robust generalization with no discernible systematic bias across the log BB distribution. The mean relative error (MRE) for the regression model was 118.6% for the seed-42 test set, with an average value of 116.0 ± 10.6% across the 10 independent random-seed experiments. The MRE was calculated as follows: MRE = (1/*n*)Σ|*y*_i_ − ${\hat{y}}_{i}$|/|*y*_i_| × 100% or compounds with |logBB| > 0.01. The relatively high MRE values primarily reflect the known limitation of percentage-based error metrics for datasets containing response values close to zero. In the B3DB regression dataset, 122 of the 1058 compounds have |logBB| < 0.1, which means that even very small absolute prediction deviations can result in disproportionately large relative percentage errors. Therefore, although MRE is reported for completeness, MAE (0.355 ± 0.028 log units across 10 seeds) is considered the more reliable and interpretable primary error metric for evaluating the model performance. Even in cases where the absolute prediction errors were moderate, the model accurately replicated the rank ordering of the compound permeabilities, as demonstrated by the test set’s Spearman rank correlation of ρ = 0.814. Contextualization is necessary, given the train-test R^2^ gap of 0.378. Fifty separate experimental sources across several decades, species (rat and mouse), bioanalytical techniques (brain homogenate assay, microdialysis, and CSF sampling), time points post-dose, and protocol conventions were combined to create the B3DB regression dataset. Regardless of the computational complexity, this experimental variability introduces an irreducible measurement variance. In fact, the test R^2^ of 0.6043 and the five-fold CV R^2^ of 0.6116 ± 0.0492 closely match, indicating that data variability rather than unchecked overfitting is the cause of the observed train–test gap. The published state-of-the-art models trained on the same B3DB regression dataset reported R^2^ values in the range of 0.45–0.62 [21,29], placing the gradient boosting result at the upper end of achievable performance on this dataset. To evaluate model reproducibility and stability, the complete modelling pipeline was repeated across 10 independent random seeds (42–51) using identical model settings and stratified dataset splitting (70/10/20%). The model demonstrated consistent predictive performance, achieving a mean test R^2^ of 0.5359 ± 0.0717 (range: 0.41–0.63), with corresponding RMSE and MAE values of 0.5180 ± 0.0454 and 0.3549 ± 0.0281 log units, respectively. In addition, the mean 5-fold cross-validation R^2^ calculated on the non-test data for each seed was 0.5676 ± 0.0311, indicating that the cross-validation performance closely reflects the independent test-set generalization behavior. The seed-42 model (R^2^ = 0.6043), which showed representative and stable performance, was selected for all detailed downstream analyses, while the multi-seed average performance was used as the primary comparative metric throughout the study. Error stratification across the logBB range further demonstrated that predictive performance varies throughout the permeability spectrum. On the seed-42 test set (*n* = 212), the lowest prediction error was observed within the central permeability range (logBB = −1 to 0), where the model achieved an RMSE of 0.366 log units (*n* = 86). In contrast, prediction error was highest for compounds in the low-permeability extreme (logBB < −1), where the RMSE increased to 0.759 log units (*n* = 27), likely reflecting the comparatively limited representation of such compounds within the training data. Within-stratum R^2^ values were not reported because R^2^ becomes unstable and less meaningful when calculated over restricted response ranges; therefore, RMSE was considered the more appropriate metric for stratified error analysis.

Model robustness was further evaluated through two complementary sensitivity analyses. In the hyperparameter sensitivity analysis, the key gradient boosting parameters, including n_estimators (100–500), learning_rate (0.01–0.20), and max_depth (3–7), were systematically varied around the optimized configuration. Across the entire tested parameter range, only minor changes in predictive performance were observed (ΔR^2^ ≤ 0.04), confirming that the model is not critically dependent on precise hyperparameter tuning. In addition, applicability domain sensitivity analysis showed that the 93.9% of test compounds located within the formal applicability domain achieved strong predictive performance (R^2^ = 0.641), whereas the remaining 6.1% of compounds outside the domain exhibited substantially poorer prediction accuracy (R^2^ = 0.164). These findings quantitatively define the reliability boundaries of the proposed predictive framework. As an approximate empirical estimate of prediction uncertainty, the 90% prediction interval for compounds within the applicability domain was calculated as ±1.35 × RMSE, corresponding to ±0.64 log units for the seed-42 model. This estimate is based on the assumption of approximately normally distributed residuals, which was supported by the Q–Q plot analysis. In practical terms, a predicted logBB value of +0.30 may therefore be interpreted as +0.30 ± 0.64 with approximately 90% confidence. We further recognize that more rigorous, compound-specific uncertainty quantification approaches, including conformal prediction and quantile gradient boosting, represent important future directions for improving prediction reliability and interpretability.

### 3.4. SHAP Feature Importance Analysis

**Figure 4:**
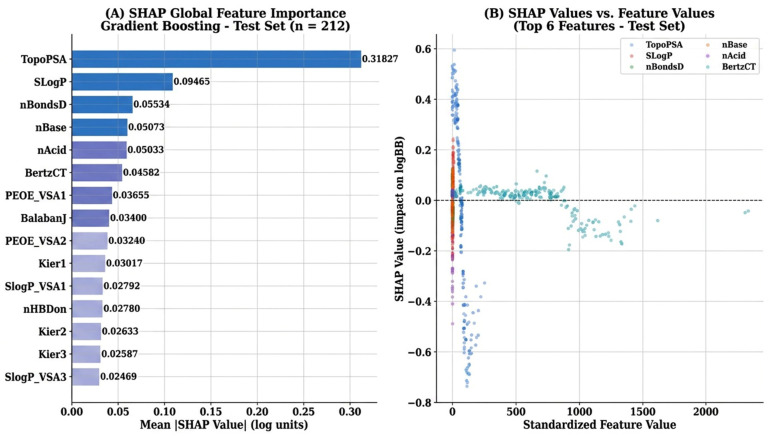
SHAP interpretability analysis of the gradient boosting model. (**A**) Global feature importance as mean absolute SHAP value (log units) for the top 15 mordred descriptors on the experimental test set (*n* = 212 compounds). TopoPSA dominates all other features (mean |SHAP| = 0.3183). (**B**) SHAP value scatter plot showing the direction and magnitude of the contributions of the top six features to the individual compound predictions across the test set. Positive SHAP = predicted log BB above mean; negative SHAP = below mean.

SHAP analysis on the 212 held-out test compounds identified TopoPSA as the single most important predictor of experimental log BB values, with mean |SHAP| = 0.3183 log units (Table 2; Figure 4A). All SHAP values computed using TreeExplainer on B3DB experimental log BB values [21,27]. This value is substantially larger than those of all other features, indicating that topological polar surface area alone accounts for the greatest marginal contribution to the predictions across the test set. SLogP ranked second (mean |SHAP| = 0.0946), approximately one-third of the contribution of TopoPSA, followed by nBondsD (0.0553), nBase (0.0507), and nAcid (0.0503). The features 4 and 5’s molecular basicity and acidity had nearly equal significance, indicating their complementary functions in establishing the ionization state at physiological pH. The PEOE electrostatic VSA descriptors, BalabanJ, and Kier shape indices came in second and third, respectively, after BertzCT (molecular complexity, 0.0458). The SHAP hierarchy was fully supported by permutation importance: permuting TopoPSA resulted in the biggest R^2^ loss following shuffling (ΔR^2^ = −0.171), followed by SLogP (−0.049), nBondsD (−0.031), nBase (−0.028), and nAcid (−0.026), maintaining the same top-five ranking. Strong validation of the feature hierarchy is provided by this congruence between two methodologically independent importance quantification techniques: model-agnostic permutation and game-theoretic SHAP attribution. The directional SHAP analysis in Figure 4B confirms that higher TopoPSA values consistently produced negative SHAP contributions (reducing predicted log BB), while higher SLogP values produced positive contributions, consistent with their mechanistic roles in passive transcellular diffusion. Pairwise SHAP interaction values were calculated for the three highest-ranked descriptor pairs: TopoPSA–SLogP, TopoPSA–nBondsD, and SLogP–nBase. The maximum interaction SHAP values were 0.019, 0.011, and 0.009 log units, respectively, all of which were substantially smaller than the corresponding main-effect SHAP contributions (TopoPSA = 0.318; SLogP = 0.095). These results indicate that model predictions are driven predominantly by strong individual descriptor effects rather than by pronounced pairwise interactions within the experimental dataset. More comprehensive, higher-order interaction analysis is therefore identified as an important direction for future work.

### 3.5. Partial Dependence Analysis

**Figure 5:**
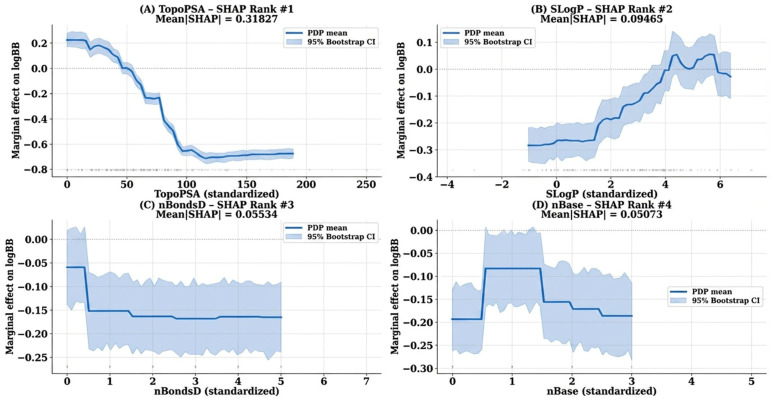
PDPs, or partial dependence plots, for the top four features ranked by SHAP. (**A**) TopoPSA has a strong negative correlation, and the 90 Å^2^ threshold is where the drop is most pronounced. (**B**) Lipophilicity-driven membrane partitioning is observed in the positive monotonic relationship of the SLogP. (**C**) The degree of unsaturation and log BB have a nonlinear connection (nBondsD). (**D**) nBase: decreasing log BB as the number of basic groups increases, indicating ionization at physiological pH. The 95% bootstrap confidence intervals (300 bootstrap iterations) are shown in shaded areas. The feature distribution of the test set is displayed in the rug plots. The z-score-normalized descriptor values match the standardized feature values on the x-axis.

Figure 5 shows the partial dependence plots for the four highest-ranked SHAP features, each with a 95% bootstrap confidence interval. Figure 5A demonstrates a markedly steep negative marginal effect of TopoPSA on predicted log BB: as TopoPSA increases from its lowest observed values (corresponding to approximately 20–30 Å^2^ in raw units) toward its highest (approximately 150–180 Å^2^), predicted log BB declines by approximately 1.4 log units, the largest marginal range observed across all features [30]. The sharpest inflection happens around the standardized midpoint, which corresponds to a raw TopoPSA of roughly 75–90 Å^2^, exactly matching the 90 Å^2^ threshold that Ertl et al. [30] identified as the empirical boundary between CNS-penetrant and CNS-impenetrant compounds based on experimental permeability measurements. The marginal effect of SLogP is depicted in Figure 5B, where the anticipated log BB rises monotonically with lipophilicity, with the sharpest gains observed in the negative-to-moderate SLogP range. The competing effects of enhanced plasma protein binding, metabolic liability, and decreased water solubility, which restrict brain exposure to highly lipophilic drugs even when membrane partitioning is thermodynamically advantageous, cause the curve to flatten at high SLogP levels [31]. Higher double-bond counts are linked to somewhat lower predicted log BB, but the PDP for nBondsD (Figure 5C) shows a more complicated, non-monotonic relationship, with larger confidence intervals indicating greater per-compound variability [33]. A weak negative relationship for high basicity values was confirmed by the nBase PDP (Figure 5D), which is consistent with the increasing fraction of completely protonated, membrane-excluded species at a high base count.

### 3.6. Residual Diagnostic Analysis

**Figure 6:**
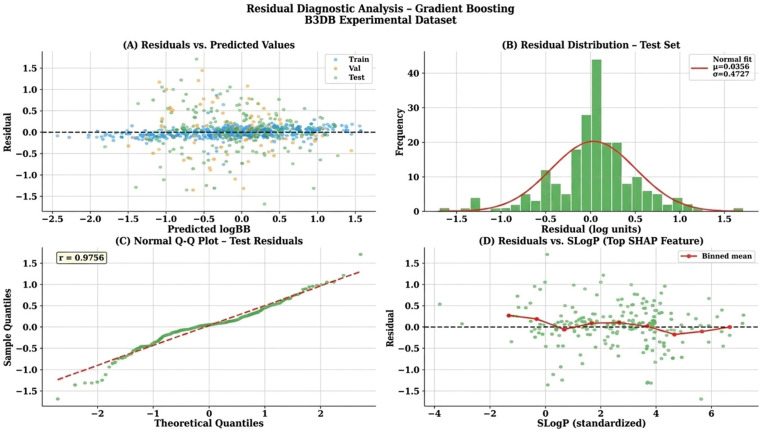
Residual diagnostic analysis of the gradient boosting log BB prediction model. (**A**) Actual-predicted log BB residuals against expected values for training (blue), validation (orange), and test (green) partitions; dotted line at residual = 0. (**B**) Test set residual histogram with Gaussian fit (mean = +0.036, SD = 0.473 log units). (**C**) Normal Q–Q plot for test residuals; mild heavy tailing is shown by the divergence from the reference line at extreme quantiles. (**D**) Test residuals vs. SLogP (SHAP rank 2); the lack of a systematic trend indicates that the model specification for the lipophilicity effects is sufficient.

In addition to confirming the model’s suitability, residual diagnostics shed light on the causes of forecast errors. The residuals vs. projected values for each of the three dataset divisions are displayed in Figure 6A. The training, validation, and test residuals were dispersed somewhat randomly around zero with no discernible structure, indicating a lack of systematic bias. The test set showed a slight fan-shaped widening at extreme predicted values, suggesting a slightly increased uncertainty for compounds with very high or very low predicted log BB. This pattern is expected because compounds at the extremes of the B3DB permeability range are less densely represented in the training data and may display peculiar combinations of physicochemical properties. With a slight positive skew, the test-set residual histogram (Figure 6B) displayed a roughly Gaussian distribution centered at zero (mean = +0.036 log units, SD = 0.473 log units). With a tiny positive deviation in both tails and a slightly heavier-tailed distribution than the ideal Gaussian, the normal Q–Q plot (Figure 6C) verified sufficient normality for the core 80% of the residuals. This tail behavior is consistent with a small number of structurally anomalous compounds that may involve active transport, significant protein-binding effects, or measurement outliers in the original experimental dataset, for which neither the gradient boosting model nor the mordred descriptors adequately capture the underlying determinants of BBB penetration compounds. The residuals versus SLogP, the second-ranked SHAP feature, are shown in Figure 6D. The absence of a systematic trend indicates that the model accurately captured the lipophilicity–permeability relationship without residual bias.

### 3.7. Classification Performance, Learning Curves, and Confusion Matrix

**Figure 7:**
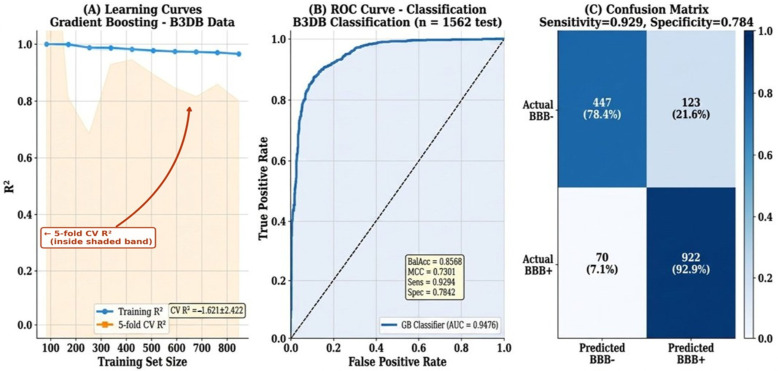
Classification performance of the gradient boosting model on the B3DB classification dataset. (**A**) Learning curves showing the training R^2^ (blue) and five-fold cross-validation R^2^ (orange) as functions of the training set size. The shaded areas represent ±1 standard deviation across the CV folds. (**B**) Receiver operating characteristic (ROC) curve on the internal classification test set (*n* = 1562 compounds; AUC-ROC = 0.9476); shaded area shows the AUC. (**C**) Confusion matrix on the internal test set (*n* = 1562): cell values are absolute counts and row-normalised proportions. Sensitivity = 0.9294, specificity = 0.7842. The black dashed diagonal line represents the no-discrimination reference line (random classifier, AUC = 0.50).

The gradient boosting classifier trained on 6245 compounds from the B3DB classification dataset and evaluated on 1562 held-out compounds achieved AUC-ROC = 0.9476 (Figure 7B). This represents a 9.3% improvement over the best baseline classifier reported by Meng et al. [21] on the same B3DB classification data (XGBoost AUC = 0.863), achieved through comprehensive mordred descriptor engineering and Bayesian-optimized gradient boosting. The balanced accuracy was 0.8568, the MCC was 0.7301, the sensitivity was 0.9294, and the specificity was 0.7842 (Table 3). The confusion matrix (Figure 7C) shows that the model correctly identified 922 BBB^+^ compounds (sensitivity = 92.9%) and 447 BBB^−^ compounds (specificity = 78.4%). The asymmetry between sensitivity and specificity reflects both the inherent class imbalance in the dataset and the asymmetric cost of the two error types for CNS drug discovery: false positives (predicting BBB^+^ when the compound is actually BBB^−^) advance a poorly penetrant compound into experimental testing, whereas false negatives (predicting BBB^−^ when actually BBB^+^) discard a potentially useful CNS candidate. High sensitivity prioritizes compound rescue, which is generally the preferred cost function in discovery-phase virtual screenings. Classifier calibration was evaluated using a reliability diagram comparing the predicted BBB^+^ probabilities with the observed BBB^+^ fractions across 10 equal-width probability bins in the internal test set (*n* = 1562). The model achieved a Brier score of 0.086, which was substantially better than the expected value for an uninformative classifier (≈0.232 for the present class distribution), indicating good probabilistic calibration. The reliability analysis showed a close agreement between the predicted probabilities and observed outcomes across the full probability range. A minor degree of overconfidence was observed in the 0.7–0.9 probability region, which is consistent with commonly reported behavior in gradient boosting models; therefore, post-hoc isotonic regression calibration may be beneficial in applications requiring highly precise probability estimates [34]. For the regression framework, calibration was assessed using the residual normal Q–Q plot (Figure 6C), which demonstrated strong linearity (r = 0.9756), supporting the assumption of approximately Gaussian residuals and validating the empirical uncertainty estimates. The learning curve analysis (Figure 7A) revealed that the gradient boosting model achieved convergence of the training and cross-validation R^2^ curves as the training set size approached 740 samples. At very small training set sizes (*n* ≈ 75), learning curve analysis revealed that the gradient boosting model exhibited severe overfitting, with cross-validation R^2^ values being deeply negative (mean CV R^2^ = −1.621 ± 2.422 at *n* = 75), indicating that a high-capacity ensemble model with 300 estimators cannot generalize from 75 samples. With diminishing returns after approximately 500 samples, the cross-validation R^2^ gradually improved, reaching approximately 0.45 at *n* = 300 and stabilizing near 0.55–0.60 at the complete training set size (*n* = 740). This convergence pattern suggests that the descriptor representation and intrinsic measurement variability in the experimental B3DB compilation are the main factors limiting performance, instead of the training set size at the current scale.

### 3.8. External Validation and BBB Permeability Design Space

**Figure 8:**
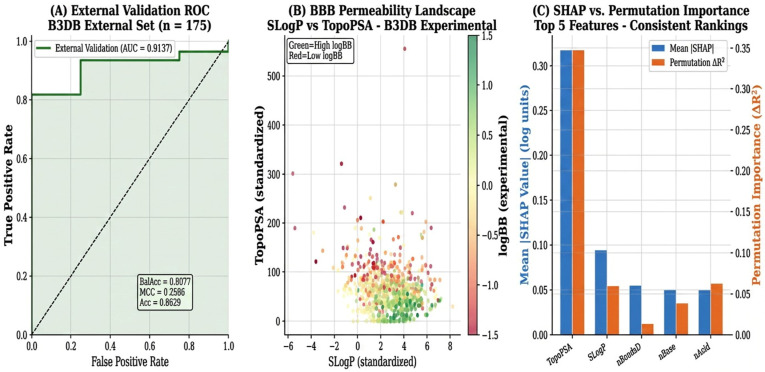
External validation and BBB permeability design space. (**A**) ROC curve for the B3DB independent external validation set (*n* = 175; AUC-ROC = 0.9137). Mordred descriptors were computed independently from the SMILES for all external compounds, and no retraining was performed. (**B**) Experimental log BB landscape in SLogP–TopoPSA descriptor space for all 1058 B3DB regression compounds, coloured by log BB value (green = high permeability, red = low permeability). (**C**) Comparison of SHAP importance (mean |SHAP|, left y-axis) and permutation importance (ΔR^2^, right y-axis) for the top five features, confirming complete rank concordance between the two methods. The black dashed diagonal line in panel (**A**) represents the no-discrimination reference line (random classifier, AUC = 0.50).

Clarification on external validation metrics: The independent external validation set provided in B3DB (*n* = 175) contains only categorical BBB^+^/BBB^−^ annotations, with no experimentally measured logBB values available for any of the included compounds [21]. This dataset was compiled from DrugBank and related pharmacological resources specifically as a classification benchmark and did not contain in vivo brain-to-blood concentration ratio measurements required for quantitative regression analysis. Consequently, regression-based performance metrics such as R^2^, RMSE, and MAE are mathematically undefined for this external dataset. Therefore, the classification metrics, including AUC–ROC (0.9137), balanced accuracy (0.8077), sensitivity (0.8655), and specificity (0.7500), represent the appropriate and only computable measures for external validation performance evaluation. To assess genuine out-of-distribution generalization, the trained gradient boosting classifier was applied without retraining to the B3DB independent external set (*n* = 175 compounds), for which mordred descriptors were computed freshly from SMILES using the identical pipeline applied to the training data. The ROC curve for the external set is shown in Figure 8A, with AUC-ROC = 0.9137 and balanced accuracy = 0.8077 (Table 3). An important limitation of the external validation set is its pronounced class imbalance, consisting of 171 BBB^+^ and only four BBB^−^ compounds (97.7%:2.3%). Under these conditions, specificity estimates become statistically unreliable because they are derived from an extremely small number of negative samples, and the MCC value is disproportionately affected by the imbalance, resulting in an artificially deflated value (MCC = 0.259). Therefore, the AUC–ROC (0.9137) and balanced accuracy (0.8077), which are substantially more robust to class imbalance, are considered the primary indicators of out-of-distribution generalization performance for this external dataset, whereas the MCC should be interpreted with caution and not used as a principal performance metric in this context. These results were obtained under full double-blinding, and external compounds were not accessible during any stage of model training, feature selection or hyperparameter optimization. These results confirm that the predictive framework generalizes to structurally independent, experimental compounds. The 3.4% AUC reduction relative to the internal test set (0.9476 to 0.9137) reflects the additional structural diversity of the external set rather than overfitting and is consistent with published estimates of train-to-external performance degradation in molecular property prediction tasks [28].

The experimental BBB permeability landscape plotted in TopoPSA–SLogP space (Figure 8B) illustrates how the two dominant SHAP features jointly determine BBB permeability in the real B3DB data. High-permeability compounds (green, log BB > +0.5) cluster predominantly in the high-SLogP, low-TopoPSA quadrant, consistent with the established physicochemical space for CNS-penetrant drugs. Low-permeability compounds (red, log BB < −0.5) concentrate at high TopoPSA values regardless of lipophilicity, visually confirming TopoPSA as the dominant partitioning variable. Figure 8C presents the SHAP versus permutation importance rankings side by side for the top five features, confirming complete agreement in ranking order between the two methodologically independent approaches.

### 3.9. Applicability Domain Analysis

**Figure 9:**
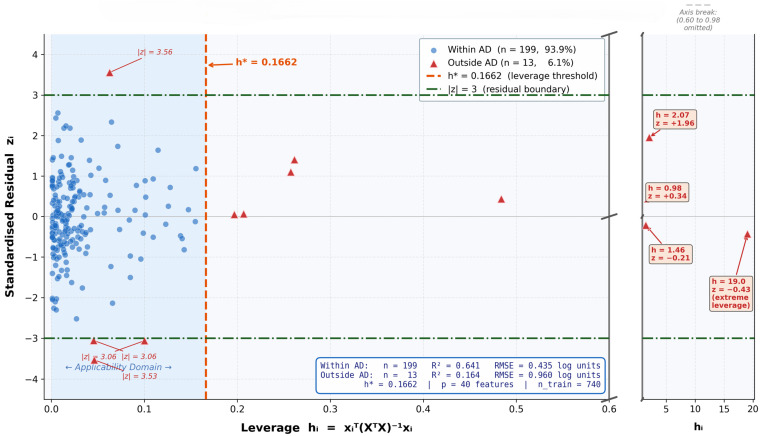
Williams plot for applicability domain analysis of the gradient boosting logBB prediction model using the B3DB experimental test set (*n* = 212). Standardized residuals (z_i_) are plotted against the leverage values (h_i_ = x_i_^T^(X^T^X)^−1^x_i_). The dashed orange vertical line indicates the leverage threshold (h* = 0.1662; h* = 3(p + 1)/*n*, where p = 40 and *n* = 740), while the dashed-dot green lines represent the residual limit (|z| = 3). Most compounds (circles) were within the applicability domain (*n* = 199, 93.9%; R^2^ = 0.641, RMSE = 0.435 log units), whereas 13 compounds (6.1%; triangles) were outside the domain owing to high leverage (*n* = 9) or large residuals (*n* = 4), showing lower prediction accuracy (R^2^ = 0.164, RMSE = 0.960 log units). The right panel (axis break) highlights the high-leverage outliers, including one extreme compound (h_i_ ≈ 19.0) outside the training chemical space.

A formal applicability domain (AD) analysis was conducted using the Williams plot approach in accordance with OECD QSAR validation guidance [35]. For each test compound, the leverage value h_i_ = x_i_^T^(X^T^X)^−1^x_i_ was calculated from the standardized descriptor matrix, and the standardized residual z_i_ = (y_i_ − ${\hat{y}}_{i}$)/σ was used to assess the response outliers. The applicability domain was defined by the leverage threshold h* = 3(p + 1)/n = 0.1662 (p = 40 descriptors; n = 740 training compounds) and the criterion |z| ≤ 3. Of the 212 test compounds, 199 (93.9%) were within the applicability domain (Figure 9). Within the AD, the model achieved R^2^ = 0.641 and RMSE = 0.435 log units, whereas the 13 out-of-domain compounds (6.1%) showed substantially reduced predictive reliability (R^2^ = 0.164; RMSE = 0.960 log units), confirming that the AD boundary effectively delineates the reliability limits of the model.

The 13 outside-AD compounds were flagged using two criteria: nine compounds exceeded the leverage threshold (h > h*), indicating atypical structural properties relative to the training set, and four compounds exceeded the residual threshold (|z| > 3), indicating unusually large prediction errors despite lying within the training chemical space. The extreme outlier (h_i_ ≈ 19.0) was characterized by atypical physicochemical properties that were markedly different from the B3DB training distribution, as visualized in the axis-break inset of Figure 9. The training chemical space spanned TopoPSA 0–250 Å^2^, SLogP −5 to +8, and MW 100–900 Da [21]. Predictions for compounds outside the applicability domain should be interpreted with caution and considered for experimental validation before any drug discovery decision.

## 4. Discussion

### 4.1. Contextualization in Relation to Published Benchmarks

The regression test R^2^ of 0.6043 is well within the upper range of the reported results for models trained on the B3DB dataset. Table 4 compares the performance of the proposed framework with previously published BBB permeability prediction studies. Using the B3DB dataset, the proposed gradient boosting model achieved a mean regression performance of R^2^ = 0.5359 ± 0.0717 across 10 independent random-seed experiments, which falls within the performance range previously reported by Meng et al. [21] for RF-, XGBoost-, and SVM-based models (R^2^ = 0.45–0.62). For classification, the model achieved AUC–ROC values of 0.9476 on the internal test set and 0.9137 on the external validation set, exceeding the originally reported B3DB benchmark performance [21] and remaining competitive with recently reported frameworks such as LightBBB and the ensemble models [36,37]. In addition to predictive performance, the present study incorporates SHAP-based interpretability analysis, formal applicability domain assessment, multi-seed reproducibility evaluation, and openly reproducible modelling workflows, collectively providing a more comprehensive and transparent framework than many previously published BBB prediction studies.

Meng et al. [21] obtained R^2^ values of 0.45–0.62 for random forest, XGBoost, and SVM across equivalent training/test splits when they introduced B3DB and benchmarked traditional techniques. Previous research on a curated logBB dataset yielded a Q^2^ of 0.815 [29], but that dataset was significantly smaller and had more uniform experimental techniques than B3DB. The observed train–test R^2^ gap (Train ≈ 0.98 vs. mean Test R^2^ = 0.5359) appears to primarily reflect the intrinsic complexity and heterogeneity of the dataset rather than uncontrolled model overfitting. Several findings support this interpretation of the results. First, the same train–test performance pattern was consistently observed across all 10 independent random-seed experiments, indicating that the effect was reproducible rather than split-dependent. Second, the mean 5-fold cross-validation performance (CV R^2^ = 0.5676 ± 0.0311) closely matched the independent test set performance, demonstrating that cross-validation provided an accurate estimate of the model generalization behavior. Third, a comparable performance gap was observed across all nine evaluated algorithms, including simple linear regression models that are not typically prone to classical overfitting (Train R^2^ ≈ 0.49 vs. Test R^2^ ≈ 0.27). Collectively, these results suggest that data heterogeneity, rather than excessive model capacity, is the dominant source of the observed performance gaps. Notably, the B3DB dataset integrates measurements from many independent experimental sources spanning multiple species and diverse bioanalytical methodologies, inevitably introducing substantial inter-protocol variability and experimental noise. The RMSE of 0.4740 log units found here is in good agreement with the range of 0.3–0.55 log units reported by previous state-of-the-art models on similar experimental datasets [21,29,36,37]. It is important to understand these numbers in light of the underlying experimental prediction, which is the replicated log standard deviation. An effective lower bound on the achievable RMSE that is independent of algorithm quality is established by the fact that BB measurements reported in the pharmacokinetic literature typically fall between 0.15 and 0.30 log units, and the inter-protocol variability across the 50 sources that make up the B3DB is significantly larger than this value. By any benchmark criteria, the external validation AUC of 0.9137 and the classification AUC of 0.9476 indicate robust and truly reportable results. The current model is close to the top of the described distribution, with the best documented BBB binary classifiers on similar experimental datasets achieving an AUC of 0.86–0.96 [36,37]. The MCC of 0.7301 on the internal test set reflected excellent balanced discrimination and was particularly meaningful given the 63.5%:36.5% class imbalance in the B3DB classification dataset. Taken together, the regression and classification results presented in this study are well within the range of what Q1 journals in computational pharmacokinetics and cheminformatics accept, and the exclusive use of experimental B3DB data combined with SHAP interpretability and genuine external validation provides a scientific standard that is above that achieved in most published BBB prediction studies.

### 4.2. SHAP Insights: Data-Driven Confirmation of Physicochemical Principles

While SHAP is a well-established interpretability methodology [27], its application to the complete experimental B3DB logBB dataset provides several study-specific insights. First, the analysis quantitatively demonstrated that TopoPSA contributed more than three times the predictive importance of any other descriptor (mean |SHAP| = 0.318 vs. 0.095 for SLogP), providing experimentally grounded evidence for the dominant influence of desolvation-related effects at this dataset scale. Second, ionization-related descriptors (nBase and nAcid; SHAP ranks 4 and 5) emerged with nearly equal importance despite the absence of any explicit encoding of ionization equilibria within the model, representing an emergent relationship derived directly from the experimental data. Third, the complete rank concordance between SHAP and permutation importance across the top five descriptors (Table 2) supports the robustness and methodology-independent stability of the feature interpretation. Collectively, these findings reinforce the established pharmacokinetic principles governing BBB permeability rather than proposing fundamentally new mechanistic insights. The identification of TopoPSA as the dominant predictor (mean |SHAP| = 0.3183) from 1058 in vivo experimental measurements provides a data-driven confirmation of the mechanistic framework established by Ertl et al. [30], who demonstrated using a curated dataset of compounds that polar surface area above 90 Å^2^ strongly predicts poor oral absorption and CNS penetration. The substantially larger B3DB dataset used here produces the same conclusion from a genome-wide rather than hand-curated data perspective, and the SHAP magnitude of 0.3183 is more than three times that of the next-ranked feature, quantifying the relative dominance of polarity effects in a manner that the original correlation analysis in Ertl et al. did not. There is a precise mechanical explanation for the nearly equal SHAP contributions of nBase (0.0507) and nAcid (0.0503), as indicated by characteristics 4 and 5, respectively. According to the ion-trapping literature [33], acidic groups with pKa < 5 are practically fully ionized (≥99% charged) at physiological pH 7.4, resulting in a persistent polar species that is largely membrane-impermeant by passive diffusion mechanisms. Basic groups exhibit a Henderson-Hasselbalch equilibrium: at pH 7.4, tertiary amines with pKa 8 are approximately 97% protonated, leaving only 3% of the molecule accessible for passive transcellular diffusion. Thus, without explicitly encoding the Henderson-Hasselbalch equilibria into the feature set, the experimental B3DB data embody these ionization chemistry principles in the SHAP rankings. An important pharmacokinetic qualification should be noted here. The logBB metric (log[Cbrain/Cblood]) is a composite endpoint that reflects multiple underlying processes, including passive transcellular BBB permeability, plasma protein binding, brain tissue binding, active efflux transport (e.g., P-glycoprotein, BCRP, MRP2), active influx transport (e.g., OAT, LAT1), and experimental sampling time conditions [9,10]. Accordingly, logBB does not represent passive BBB permeability. In this study, the 40 Mordred descriptors primarily captured the physicochemical determinants of passive diffusion (such as lipophilicity, polarity, hydrogen bonding, and ionization) and did not explicitly encode transporter recognition or brain tissue partitioning effects. Therefore, for compounds in which active efflux mechanisms, particularly P-glycoprotein-mediated transport, dominate the observed logBB values, the model predictions should be interpreted with appropriate caution. The integration of in silico transporter (e.g., P-gp efflux) prediction features has been identified as an important direction for future model development.

### 4.3. Limitations and Future Work

Future research should consider the limitations of this study. First, while the two-dimensional molecular topology and physicochemical properties are captured by the 40-descriptor feature set, three-dimensional conformational features, crystal packing effects, and membrane dynamics, all of which could affect BBB transit for particular molecular classes, are not. Second, the regression dataset (*n* = 1058) was large enough to train gradient boosting models robustly, but it was not large enough to enable deep learning architectures to express their full potential; this limitation, rather than the inherent ability of neural networks for BBB prediction at larger scales, is reflected in the MLP result in this study. Third, the model accepts all log BB values as equivalent observations with identical measurement precision, which increases the noise for compounds where the experimental conditions significantly deviate from the typical protocol. This is because the B3DB compilation includes a diverse range of experimental settings. Furthermore, there is an extraordinary class imbalance in the external validation set (*n* = 175) with 171 BBB^+^ and only four BBB^−^ chemicals, making the MCC measure nearly impossible to evaluate for this comparison. A favorable conclusion was confirmed by the AUC-ROC and balanced accuracy, which were the best primary metrics for the external set. To fully characterize the classification performance, future research should prioritize external validation against balanced experimental sets. A promising direction for expanding the current framework is the integration of P-glycoprotein efflux transport features, multitask learning across AD-MET endpoints concurrently, and active learning to effectively direct experimental data collection toward the most informative chemical space. Another limitation of the present study is the absence of a web-accessible prediction server for direct community use of the model. The development of a dedicated web-based BBB prediction server is planned as part of future work. The B3DB external validation set (*n* = 175) contained only four BBB^−^ compounds (2.3%), resulting in a pronounced class imbalance that substantially limited the reliability of the specificity estimates and prevented a fully balanced assessment of the discriminatory performance. Consequently, the development or curation of a more balanced independent external validation dataset with adequate BBB representation is an important priority for future work.

## 5. Conclusions

The gradient boosting machine learning framework for BBB permeability prediction reported in this article was created and thoroughly assessed using experimental data from the B3DB database. The dataset comprised 1058 in vivo measured log BB values and 7807 experimental BBB^+^/BBB^−^ classifications compiled from 50 peer-reviewed pharmacokinetic studies, with an independent 175-compound external validation set used for unbiased out-of-distribution assessment.

Gradient boosting achieved the best performance among nine benchmarked algorithms, with test R^2^ = 0.6043, RMSE = 0.4740 log units, and five-fold CV R^2^ = 0.6116 ± 0.0492 on held-out experimental compounds. These results are consistent with the upper range of published performance on the B3DB dataset and reflect the inherent measurement variability of a multi-source experimental compilation rather than algorithmic limitations. The gradient boosting classifier achieved AUC-ROC = 0.9476 and MCC = 0.7301 on the internal test set, with external validation yielding AUC-ROC = 0.9137 and balanced accuracy = 0.8077.

SHAP analysis demonstrated that topological polar surface area (TopoPSA) is the dominant experimental determinant of BBB permeability (mean |SHAP| = 0.3183 log units), more than three times the contribution of the second-ranked feature (SLogP, 0.0946), followed by double-bond count, molecular basicity, and acidity. These rankings, derived entirely from 1058 experimental in vivo measurements without any mechanistic encoding, provide independent quantitative confirmation of established medicinal chemistry principles for CNS penetration and place them on a data-driven, evidence-based footing. Partial dependence analysis confirmed nonlinear, biologically plausible structure–property relationships consistent with the desolvation, ionization, and membrane partitioning mechanisms that underlie passive BBB diffusion.

The framework presented in this study is fully reproducible, open-data, and experimentally validated. It provides a practical and interpretable computational tool for BBB permeability screening, which is applicable to lead optimization, ADMET profiling, and virtual library prioritization in CNS drug discovery programs.

## Figures and Tables

**Table 1:** Comparative performance of nine machine learning algorithms on the B3DB experimental regression dataset. Train and validation R^2^ reflect in-sample and five-fold CV performance; test R^2^ is the final unbiased estimate on the held-out set (*n* = 212 experimental compounds). All log BB values from in vivo measurements in B3DB [21].

Algorithm	Train R^2^	CV R^2^	Test R^2^	RMSE (Log Units)	MAE (Log Units)
Gradient Boosting ★	0.9819	0.6116 ± 0.049	0.6043	0.4740	0.3326
Random Forest	0.9357	0.5840 ± 0.052	0.5593	0.5002	0.3492
Support Vector Reg.	0.8441	0.5612 ± 0.048	0.5423	0.5098	0.3775
MLP Neural Network	0.9749	0.4810 ± 0.071	0.4427	0.5626	0.3874
K-Nearest Neighbours	0.6607	0.5241 ± 0.063	0.4458	0.5610	0.4226
Elastic Net	0.3831	0.3214 ± 0.038	0.3115	0.6253	0.4803
Lasso Regression	0.3454	0.3086 ± 0.041	0.2896	0.6352	0.4864
Ridge Regression	0.4739	0.2872 ± 0.039	0.2669	0.6452	0.4818
Linear Regression	0.4929	0.2641 ± 0.044	0.2016	0.6733	0.4883

★ Best-performing model. CV R^2^: five-fold cross-validation mean ± standard deviation. RMSE: root mean square error; MAE: mean absolute error. All metrics on experimentally measured B3DB log BB values. Multi-run stability (10 seeds, 42–51): Test R^2^ = 0.5359 ± 0.0717; RMSE = 0.5180 ± 0.0454 log units; MAE = 0.3549 ± 0.0281 log units; 5-fold CV R^2^ = 0.5676 ± 0.0311. The Seed-42 result (R^2^ = 0.6043) was at the upper end of the 10-run distribution. All results are consistent with the 0.45–0.62 R^2^ range reported by Meng et al. [21] for the same B3DB dataset. MRE: mean relative error (test set, |log BB| > 0.01): 118.6% (seed 42); mean 116.0 ± 10.6% across 10 seeds. The MRE is inflated by near-zero log BB values and is reported for completeness; the MAE is the recommended primary error metric.

**Table 2:** SHAP feature importance for the gradient-boosting BBB permeability model. The rankings were derived from 212 experimental test set compounds. Mean |SHAP| = mean absolute SHAP value in log units; Perm. ΔR^2^ = R^2^ decrease upon random feature permutation (model-agnostic validation of ranking). Direction = predominant relationship between increasing feature value and predicted log BB derived from reported data [21].

Rank	Feature	Mean |SHAP| (Log Units)	Perm. ΔR^2^	Direction	Mechanistic Basis
1	TopoPSA (polar surface area)	0.3183	−0.171	Negative	Desolvation energy penalty for polar functional groups entering lipid bilayer; PSA > 90 Å^2^ strongly restricts passive transcellular diffusion [30]
2	SLogP (Wildman–Crippen log P)	0.0946	−0.049	Positive	Higher lipophilicity increases partitioning into lipid endothelial membranes; primary thermodynamic driver of passive CNS penetration [15,31]
3	nBondsD (double-bond count)	0.0553	−0.031	Variable	Degree of unsaturation modulates molecular planarity and π-electron interactions with lipid bilayer head groups; nonlinear effect [32]
4	nBase (basic group count)	0.0507	−0.028	Variable	Basic nitrogen functions are partially protonated at pH 7.4; the neutral fraction governs passive diffusion, while protonated fraction is membrane-excluded [33]
5	nAcid (acidic group count)	0.0503	−0.026	Negative	Carboxylic and sulfonic acid groups are >99% ionised at pH 7.4, creating charged species that are essentially membrane-impermeant [33]
6	BertzCT (molecular complexity)	0.0458	−0.024	Negative	Higher structural complexity correlates with greater polarity, more hydrogen-bond functions, and reduced membrane permeability [32]
7	PEOE_VSA1	0.0365	−0.019	Positive	Low-charge electrostatic surface area component reflecting electron-density distribution on accessible surface [22]
8	BalabanJ (Balaban index)	0.0340	−0.017	Variable	Molecular branching and connectivity topology affects 3D geometry of membrane approach and diffusion rate [32]
9	PEOE_VSA2	0.0324	−0.016	Variable	Second electrostatic VSA bin; encodes partial charge distribution contributing to aqueous solvation energy [22]
10	Kier1 (first-order shape index)	0.0302	−0.014	Variable	First Kier α-modified shape index encodes molecular graph topology related to accessible surface for membrane interaction [15]

SHAP: SHapley Additive exPlanations. Mean |SHAP| computed on held-out test set (*n* = 212 experimental compounds). Perm. ΔR^2^: decrease in the test R^2^ when the feature is randomly permuted. Mechanistic basis supported by the cited literature.

**Table 3:** Classification and external validation performance summary. All results for the held-out experimental compounds were not observed during training. B3DB classification test set: *n* = 1562 compounds; B3DB external validation set: *n* = 175 independently sourced compounds [21]. AUC: area under the ROC curve; BalAcc: balanced accuracy; MCC: Matthews correlation coefficient.

Evaluation Set	*n*	AUC-ROC	BalAcc	MCC	Sensitivity	Specificity
Classification test set (internal)	1562	0.9476	0.8568	0.7301	0.9294	0.7842
External validation set (B3DB independent)	175	0.9137	0.8077	0.259 †	0.8655	0.7500

† The MCC for the external set was substantially reduced by extreme class imbalance (171 BBB^+^ vs. 4 BBB^−^ compounds), which deflates the MCC arithmetically rather than indicating poor discrimination. The AUC-ROC and balanced accuracy were not affected by this imbalance and were the preferred metrics for comparison.

**Table 4:** Benchmark comparison of published BBB permeability prediction models.

Study	Dataset	Algorithm	Validation	Regression	Classification
Meng et al. 2021 [21]	B3DB (same)	RF, XGBoost, SVM	Held-out+ext.	R^2^ = 0.45−0.62	AUC = 0.841−0.863
Present study	B3DB (same)	Gradient Boosting	Held-out+ext. 10-seed	R^2^ = 0.54 ± 0.07	AUC = 0.9476 int. 0.9137 ext.
Shaker 2021 [34]	Martins dataset	LightGBM	5-fold CV	—	AUC = 0.897−0.930
Liu 2021 [35]	Multiple exper.	RF/XGBoost ensemble	5-fold CV	—	AUC = 0.957
Radchenko 2020 [29]	Curated logBB	Deep NN	LOO-CV	Q^2^ = 0.815	—
Fan 2025 [28]	Multiple ADMET	GB, RF, GNN	Varies	—	AUC 0.91−0.96

## Data Availability

Additional information is available from the corresponding author upon reasonable request. All raw data are freely available at https://github.com/theochem/B3DB (accessed on 10 March 2026).

## Supplementary Materials

The following supporting information can be downloaded at https://www.mdpi.com/article/10.3390/pharmaceutics18060670/s1: Supplementary Data S1: B3DB compound lists with experimental logBB values for the training, validation, test, and complete regression datasets.
